# Contrasting responses to climate change at Himalayan treelines revealed by population demographics of two dominant species

**DOI:** 10.1002/ece3.5968

**Published:** 2020-01-28

**Authors:** Kumar Mainali, Bharat Babu Shrestha, Ravi Kumar Sharma, Arjun Adhikari, Eliezer Gurarie, Michael Singer, Camille Parmesan

**Affiliations:** ^1^ Department of Integrative Biology The University of Texas at Austin Austin TX USA; ^2^ Department of Biology University of Maryland College Park MD USA; ^3^ Central Department of Botany Tribhuvan University Kirtipur Kathmandu Nepal; ^4^ Department of Biology Nobel Academy Higher Secondary School and College Kathmandu Nepal; ^5^ Department of Natural Resource Ecology and Management Oklahoma State University Stillwater OK USA; ^6^ School of Biological Sciences Plymouth University Plymouth UK; ^7^ Theoretical and Experimental Ecology (SETE) UMR 5321 CNRS University Paul‐Sabatier Moulis France; ^8^ Department of Geological Sciences The University of Texas at Austin Austin TX USA

**Keywords:** *Abies spectabilis*, climate change, global warming, Nepal Himalaya, *Rhododendron campanulatum*, treeline shift

## Abstract

1. Alpine treelines are expected to shift upward due to recent climate change. However, interpretation of changes in montane systems has been problematic because effects of climate change are frequently confounded with those of land use changes. The eastern Himalaya, particularly Langtang National Park, Central Nepal, has been relatively undisturbed for centuries and thus presents an opportunity for studying climate change impacts on alpine treeline uncontaminated by potential confounding factors.
2. We studied two dominant species, *Abies spectabilis (AS)* and *Rhododendron campanulatum (RC)*, above and below the treeline on two mountains. We constructed 13 transects, each spanning up to 400 m in elevation, in which we recorded height and state (dead or alive) of all trees, as well as slope, aspect, canopy density, and measures of anthropogenic and animal disturbance.
3. All size classes of *RC* plants had lower mortality above treeline than below it, and young *RC* plants (<2 m tall) were at higher density above treeline than below. *AS* shows little evidence of a position change from the historic treeline, with a sudden extreme drop in density above treeline compared to below. Recruitment, as measured by size–class distribution, was greater above treeline than below for both species but *AS* is confined to ~25 m above treeline whereas *RC* is luxuriantly growing up to 200 m above treeline.
4. *Synthesis*. Evidence suggests that the elevational limits of *RC* have shifted upward both because (a) young plants above treeline benefited from facilitation of recruitment by surrounding vegetation, allowing upward expansion of recruitment, and (b) temperature amelioration to mature plants increased adult survival. We predict that the current pure stand of *RC* growing above treeline will be colonized by *AS* that will, in turn, outshade and eventually relegate *RC* to be a minor component of the community, as is the current situation below the treeline.

## Graphical abstract

In the Himalayan treelines of Central Nepal, *Abies spectabilis* shows little evidence of a position change from the historic treeline, whereas *Rhododendron campanulatum* is luxuriantly growing upto 200 m above treeline. Evidence suggests that the elevational limits of *R. campanulatum* have shifted upward both because of facilitation to young plants above treeline by surrounding vegetation and increased adult survival due to a general temperature amelioration to mature plants. We predict that the current pure stand of *R. campanulatum* growing above treeline will be colonized by *A. spectabilis* that will, in turn, outshade and eventually relegate *R. campanulatum* to be a minor component of the community, as is the current situation below the treeline.

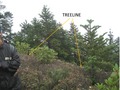

## 1 INTRODUCTION

Treeline, defined here as the upper elevational boundary of trees existing in clumps (Körner & Paulsen, 2004), essentially marks the position of a threshold temperature regime for successful recruitment and upright tree growth. Globally, both latitudinal and elevational positions of treelines are largely influenced by temperature (Cogbill & White, 1991; Jobbágy & Jackson, 2000; Jump, Mátyás, & Peñuelas, 2009; Körner, 2012; Körner & Paulsen, 2004). In analysis of year‐round temperature data from many treelines, the metric that best‐predicted alpine treeline position was a growing season mean air temperature of 6.4°C, with a minimum season length of 94 days (Paulsen & Körner, 2014), which turned out to be surprisingly similar at treelines across bioclimatic regions (Körner, 2012; Paulsen & Körner, 2014). The conclusion from this approach of identifying the invariant factor determining a hard boundary for treeline position is supported by another global analysis, which found that diffuse treelines of the world most frequently responded to increasing growing season temperature (Harsch & Bader, 2011). The prevailing view of temperature‐controlled treeline position is also supported by historic fluctuation of treeline position with temperature changes (Lloyd & Graumlich, 1997; Tinner, 2002).

Treelines are therefore expected to be responsive to current climate warming. As expected, a global meta‐analysis of treeline dynamics showed that the majority of treelines have been advancing upward or poleward (Harsch, Hulme, McGlone, & Duncan, 2009). However, treeline advance was not observed in every site. Possible reasons for this are several: (a) climate change in a local site may not necessarily follow the mean global trends (Körner, 2012); (b) the most dominant factor controlling treeline position may not be climate but disturbance‐related, such as natural disturbance forming canopy openings (Cullen, Stewart, Duncan, & Palmer, 2001) or fires mediated by El Niño‐Southern Oscillation (Brown & Wu, 2005); (c) finally, even when treelines are truly climate‐limited, there may be a lag in tree response to climate warming (Kullman, 1993; Lloyd, Rupp, Fastie, & Starfield, 2002).

Some treelines that have been changing rapidly have done so under the influence of nonclimatic anthropogenic drivers. For example, the most detailed studies of montane range shifts apparently induced by current climate change are from Europe. However, in Europe, effects of climate change are almost always confounded with those of land use change. Until the mid‐19th century, the observed treelines in the European Alps were forced downward by forest clearing to create pastures for domestic animal grazing and haymaking (Holtmeier, 2009). Hence, it is not a surprise that most of the recent upward montane vegetation shift in the European Alps is not a response to climate change. Gehrig‐Fasel, Guisan, and Zimmermann (2007) identified only 4% of new forest above the climatic treeline, while all remaining reforestation was of historically forested lands. Thus, human influences were the main driver of upward shifts of montane forests in Europe. In contrast, climatic treelines in undisturbed areas of the Canadian Rockies showed a trend toward upward shift. This upward shift is more complex than simple climate change, as fire frequency and intensity were also found to be significant drivers (Luckman & Kavanagh, 2000). In North America, shifts toward hotter, more frequent wildfires has been linked to climate change (Kirchmeier‐Young, Gillett, Zwiers, Cannon, & Anslow, 2019; Mansuy et al., 2019).

In both the Canadian and European studies, it has been difficult to dissect out separate effects of regional warming from other, more direct, anthropogenic influences. Predictions of dramatic effects of climate change on high altitude species, together with the importance of these species as early‐warning systems, warrant new studies in mountain regions in which the influence of confounding anthropogenic factors can be minimized. Large parts of the Himalaya are free from such a history of large‐scale anthropogenic effects at treelines, and so present an opportunity to explore responses of natural treelines to climate change. These responses are likely to be relatively strong in the Himalaya, which has been warming faster than surrounding lowland areas, with this trend expected to continue (IPCC, 2007, 2014). These observations make it clear that rapid change is occurring in the region, yet our understanding about impacts on biodiversity and on underlying biological processes is very limited, in spite of the region being a designated biodiversity hotspot containing 3,160 endemic plant species (Mittermeier et al., 2005).

A treeline responsive to recent climate warming has a number of characteristics including gradual upward shift of the treeline (Harsch et al., 2009), new surge of growth at the edge of distribution (Körner, 2012), high rate of recruitment and high potential for regeneration (Motta & Nola, 2001), and faster growth of individuals (Paulsen, Weber, & Körner, 2000). As the temperature regime at treeline becomes more conducive for recruitment and growth, increasingly higher numbers of seedlings and saplings are expected to survive and grow, resulting in accelerated regeneration above treeline. However, the mere presence of young recruits at one point in time must not be taken as successful recruitment, neither must the absence of seedlings be interpreted as a recruitment limitation. Such situations may be ephemeral. To maintain or advance a treeline, it needs only one or two successful waves of recruits per century. A more powerful tool to assess long‐term recruitment is tree demography. The age–class or size–class distribution helps to identify past regeneration waves of a forest (Hofgaard, Dalen, & Hytteborn, 2009; Körner, 2012). A long‐sustained regeneration pattern that is evident in tree demography, together with other evidence, can indicate a true response of the treeline ecotone to recent climate change.

For this study, within the eastern Himalaya ecoregion, we selected Langtang National Park (LNP), Central Nepal. The LNP region has been relatively undisturbed for centuries at treeline elevation. Regulated grazing by nomadic herders has been ephemeral and infrequent, and other human activities have had even lower impacts. Firewood is collected, both by the herders who tend to stay in a site for about a month, and by two or three small tourist stations that operate for half of each year and receive few customers. Collectively, these human activities are unlikely to have altered the position of the treeline.

The substantial warming in the high Himalaya, coupled with an expected high sensitivity of montane species to climate change, provides an excellent arena for studying responses to climate change by plants in the vicinity of treeline. The objectives of this study were to examine the population structure of the two most common species near the treeline in central Nepal and to identify patterns and predictors of reproduction, survival, and abundance of these species across elevational gradients that include treeline. We then used this information to (a) determine the regeneration potential of each species along the elevational gradient and (b) examine whether an upward shift of either or both species has occurred in recent decades.

## 2 METHODS

### 2.1 Definitions

In the context of the current work, we define the life form “tree” following Körner (2012) as an upright, single‐stemmed woody plant that is tall enough to experience the conditions of the free atmosphere. In contrast, tree seedlings exist in the aerodynamically sheltered boundary layer of grasses and dwarf shrubs which are thermally decoupled from atmospheric circulation and warmer than ambient air during sunshine hours. Saplings hold an intermediate position, and depending on the height of surrounding vegetation and topography, they may transit from the warmer ground layer to the cooler free air. Therefore, the absolute height is not an exhaustive criterion for a plant to experience its environment as a tree, a sapling or a seedling, but needs to be expressed relative to the surrounding vegetation. The loss of aerodynamic shelter commonly occurs between 1 and 3 m height. In our case, with a dwarf shrub ground cover, we used 2 m height as a criterion for defining a tree individual of *Abies spectabilis*.

**Figure 1:**
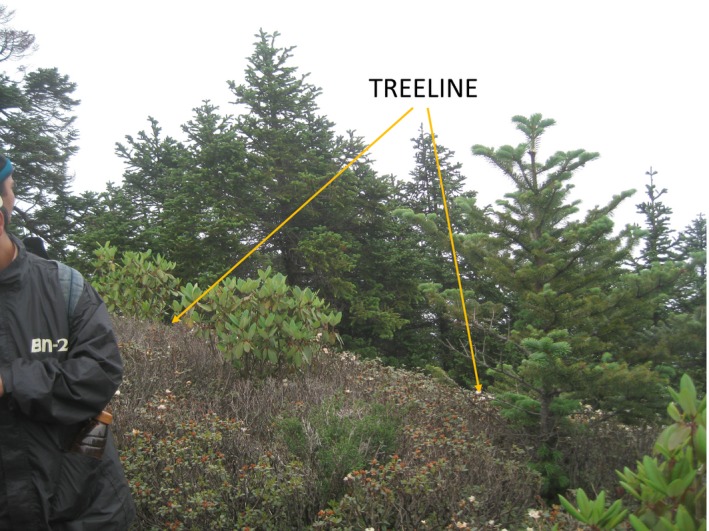
Treeline in site 2 with sharp boundary of tall *Abies spectabilis*. Isolated individuals of *A. spectabilis* and *Rhododendron campanulatum* are seen in the space above treeline which supports thick ground vegetation (0.5–1 m tall, generally). Above the treeline, tree or nontree individuals of *Abies* are found in solitary. *Rhododendron*, on the contrary, makes dense and pure clusters, growing luxuriantly up to 200 m above treeline (not seen in this picture)

Following Körner and Paulsen (2004), we defined the uppermost boundary of the closed forest as *timberline*. Above the timberline, the forest is open, with trees found in groups at higher elevation. The virtual line that connects these uppermost tree groups is called *treeline*. The uppermost boundary at which any individual of a species (of any size or class) is found is defined as the tree species line (the *species limit*). The *treeline ecotone* refers to the space between timberline and the species limit (Körner, 2012). It is a transition zone at the upper elevational boundary of the life form tree where individuals rapidly change their physiognomy, giving way to treeless alpine terrain upslope. We describe our measures and observations with respect to the very conspicuous treeline defined by *Abies spectabilis* (Figure 1) that grows to a height of >10 m right at the treeline.

### 2.2 Study sites

Our study sites are in Nepal's Langtang National Park, which covers 1,710 km^2^ area with an elevation ranging from 1,000 to 7,200 m (Fox, Yonzon, & Podger, 1996). Situated about 45 km north of Kathmandu valley, the park provides areas for grazing for nearby villages. However, our field sites, located at the treeline ecotone, are distant from human settlements. One of our two study sites (Site 1) is two hours' walk from the nearest tourist trail, which itself is one of the least preferred tourist trails. The second study site (Site 2) is much more isolated. The two sites were both situated on north‐facing slopes, 4 km apart. On each mountain, there was considerable area above the treeline with a thick cover of alpine meadow and dwarf shrubs as well as small individuals of various tree or shrub species, either in isolation or in groups (*Betula utilis*, *Sorbus microphylla, Salix* sp.). The area above treeline varied in size but always encompassed at least 300 m elevational span. The highest elevations with vegetation had no individuals of tree species, not even small saplings.

As expected from reported regional trends (IPCC, 2007, 2014), the climate at our study sites has indeed been warming: using gridded monthly estimates by Climate Research Unit (TS3.10, 0.5° spatial resolutions), Mitchell and Jones (2005) show that the area has been warming since *ca*. 1970, whereas the long‐term trend of precipitation has remained stationary (Shrestha, Hofgaard, & Vandvik, 2014). Weather data collected in a Kathmandu station (the nearest one with complete long‐term data, ~50 km south from the field sites) indicate a regional warming trend in both annual and seasonal (winter, spring, and summer) temperatures (Shrestha et al., 2014). IPCC (2007, 2014) report more frequent warm nights in the region and a decline in cooler weather, with an increase in variability of precipitation.

### 2.3 Focal species

We studied the two most abundant tree or woody tall shrub species in the treeline ecotone of our study sites, *Rhododendron campanulatum* D. Don, locally known as *chimal*, and Himalayan silver fir,* Abies spectabilis* D. Don, locally known as *gobre salla*, *talis patra*, and *thingre salla*. *Abies spectabilis* is the tallest species locally, and one of the most abundant evergreen conifers. It grows in a wide elevational belt of 2,800–4,000 m in the Himalaya (Polunin & Stainton, 1984). The tree has a single main axis holding whorls of branches, each of them profusely branched and densely foliated. The treeline, typically comprised of tall, old trees of *A. spectabilis*, generally marks the most conspicuous upper boundary of tree individuals in the central Nepal Himalaya (Figure 1). In the study sites and in other upper distributional limits, *A. spectabilis* was very often associated with *R. campanulatum*, an abundant evergreen broad‐leaved woody shrub, sometimes classified as a tree, achieving heights of 2–6 m, and distributed between 3,000 and 4,400 m (Polunin & Stainton, 1984). When *R. campanulatum* grows as understory vegetation under the canopy of *A. spectabilis*, it makes much less dense vegetation than it does in the absence of trees that shade it. *Rhododendron campanulatum* can also form very dense thickets that may lift the aerodynamic boundary to a couple meters aboveground. This means that *R. campanulatum* may still be profiting from the sheltered climate near the ground at a height at which *Abies* is already fully exposed to convective heat transfer. This awaits a verification by on‐site microclimatic studies. We thus decided to describe the *Rhododendron* bush above the *Abies* treeline as “tall shrub canopy,” the limit of which (for >2 m tall individuals) may be up to 200 m above the climatic tree limit.

We recorded abundances of all tree species found in our plots in the treeline ecotone. In descending order of abundance, these species were as follows: *R. campanulatum*, *A. spectabilis*, *Salix* sp., *Sorbus microphylla*, *Betula utilis*, and *Prunus* sp. Their densities in the study sites, listed in the same order of abundance were as follows: 5,751, 1,374, 588, 418, 266, and 48 per hectare, respectively. In the current study, we analyzed only data for *R. campanulatum* and *A. spectabilis*.

### 2.4 Experimental design and measurements

We set up plots along 13 elevational transects (up to eight plots per transect) that crossed the treeline. Plots of size 10 m × 10 m were spaced 50 m apart in elevation. The plot censuses recorded heights and state (dead or alive) of all individuals inside the plots. We also measured a range of potential covariates at each plot: steepness and aspect of slope, and canopy cover created by adults. We used number of cut trees/stumps (which were scarce at site 1 and absent from site 2) as a proxy for anthropogenic disturbance. Slope and aspect were measured in degrees. Aspect affects plants primarily by controlling sunlight duration. For that reason, an aspect of 60° is similar in effect to 300°. Therefore, we calculated the biologically meaningful aspect from the original measurement (0–360°) as follows: use aspect as measured if <180°, subtract it from 360 if the measured aspect is 180–360°.

Mortality for a given size class in a plot was estimated as the fraction of all individuals in the size class that were dead. No stunted plants were observed inside the plots. Outside of our plots above treeline, we rarely found plants with stunted growth and they were easily distinguished from young saplings of similar size by physiognomy. Plots without any plants contributed toward estimating density but not mortality.

In some of the transects, we could not set up an equal number of plots on the two sides of treeline for the reasons of topography or lack of any vegetation. This resulted into a situation when plots on one side were further away from treeline compared to the plots on the other side. Hence, in the analysis that aggregates the response variable without relating to the explanatory variable (Figures 4 and 6), we discarded some of the plots to maintain equal number of plots on the two sides of treeline. In other analysis (e.g., regression, or for scatter plot), we included all plots.

A random subset of individuals of *R. campanulatum* above the treeline, not necessarily within the studied plots, was chosen for determining age–height–basal area relationship. We obtained a disk of the trunk at ground level. All of our samples had a single main axis at ground level. Age of the plants was determined using tree‐ring analysis at the Dendro Lab of the National Academy of Science and Technology in Kathmandu, Nepal. High resolution (300 dpi) photographs of stem disks were analyzed in Adobe Photoshop CS 5.5 for determining basal area and circumference.

Plant age distributions can be used as a proxy for rate of successful regeneration. In this study, our coring data allow us to convert plant size to age, granted that we excluded the few stunted individuals found in the entire study area. Thus, we estimated regeneration potential of the populations above and below treeline with the aid of statistics that describe the distribution of plant size. The most widely used of such measures is the Gini coefficient (*G*), which provides an estimate of inequality in distribution by measuring the area under the line of equality in the Lorenz curve (Gini, 1912; Lorenz, 1905). A smaller value of *G* indicates less inequality between the different categories, in this case different plant sizes. This statistic has long been used to understand plant populations (Bendel, Higgins, Teberg, & Pyke, 1989; Weiner & Solbrig, 1984). Although *G* is a widely used index, it cannot be used to determine the source of inequality. Two Lorenz curves with identical *G* can have different shapes, indicating different impacts of small or large individuals resulting in the same inequality index. To identify the source of inequality, Damgaard and Weiner (2000) proposed the use of another index of inequality—the Lorenz asymmetry coefficient (*S*). For a given *G*, if *S* > 1, the inequality is driven by very few large individuals that are very tall. For the same *G*, if *S* < 1, inequality is primarily due to relatively higher frequency of small plants compared to when *S* > 1 (Damgaard & Weiner, 2000). Hence, a more extreme value of *S*, either high or low, represents a situation of increased inequality. For this reason, we use both *G* and *S* in our analyses, in addition to a standard skewness coefficient or moment skewness (*g*_1_).

### 2.5 Analysis

Groups were compared with analysis of variance. For determining the predictors of abundance and mortality, generalized linear mixed models (GLMM) were created with transect as random effect and up to five fixed effects. The variables were scaled with their standard deviation for an easy comparison of the models. The age structure of a population was analyzed with three measures of inequality in the Lorenz curve, that is, skewness coefficient, Gini coefficient, and asymmetric index. All models were evaluated for their statistical significance against the acceptable false‐positive rate of 5%. Data analysis and plotting were performed in R ×64 3.0.2 (The R Project for Statistical Computing) using the following packages: e1071, fields, graphics, grDevices, grid, ineq, mvpart, plotrix, plyr, reshape, and sciplot.

## 3 RESULTS

### 3.1 Treeline position

The treeline is highly conspicuous (Figure 1) and appears to be virtually unchanged for at least a century; the 50 m elevational band immediately below treeline in the study area had *A. spectabilis* trees with an average height of 17.5 m and a mean age of 86 years (ranging from 35 to 170 years, K. Mainali, unpublished). *Abies spectabilis* individuals right at the boundary of treeline were >10 m tall. The elevation of the treeline averaged 3,856 m across the sites.

### 3.2 Elevational patterns of density and mortality

**Figure 2:**
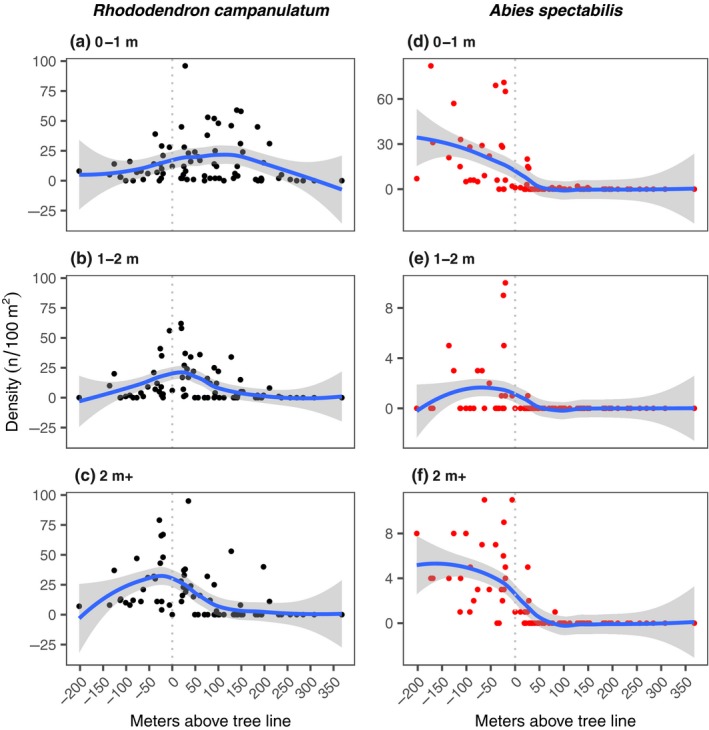
Elevational pattern of density of *R. campanulatum* (left panels) and *A. spectabilis* (right panels). Each dot in the figure represents a quadrat. The values are plotted against elevation with reference to treeline position (elevation of a plot minus elevation of treeline). The density is shown for various size classes of *R. campanulatum*: (a) 0–1 m, (b) 1–2 m, and (c) >2 m tall, and of *A. spectabilis*: (d) 0–1 m, (e) 1–2 m, and (f) >2 m tall. Smoothed pattern (thick line) and the confidence interval (band of shade) are displayed

**Figure 3:**
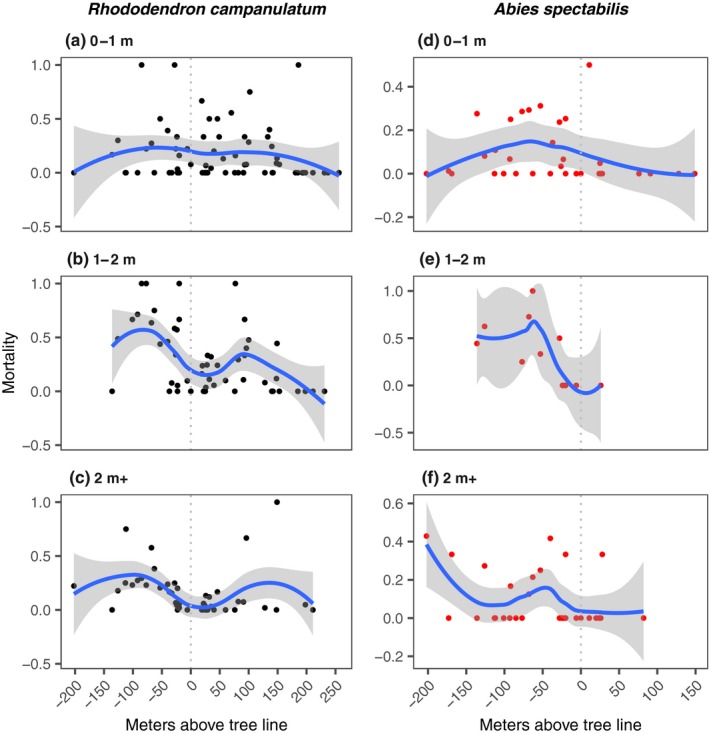
Elevational pattern of mortality (fraction of all individuals of the size class in a quadrat that are dead) of *R. campanulatum* (left panels) and *A. spectabilis* (right panels). Description as in Figure 2

*Rhododendron campanulatum* and *A. spectabilis* showed a very different pattern of density, mortality, and age structure. Densities of *R. campanulatum* tended to peak around treeline for tall plants, ~50 m above treeline for medium size plants (1–2 m height) and ~125 m above treeline for small plants (<1 m height) (Figure 2a–c). Immediately on either side of the treeline, mortality of *R. campanulatum* remained fairly high (Figure 3a–c).

**Figure 4:**
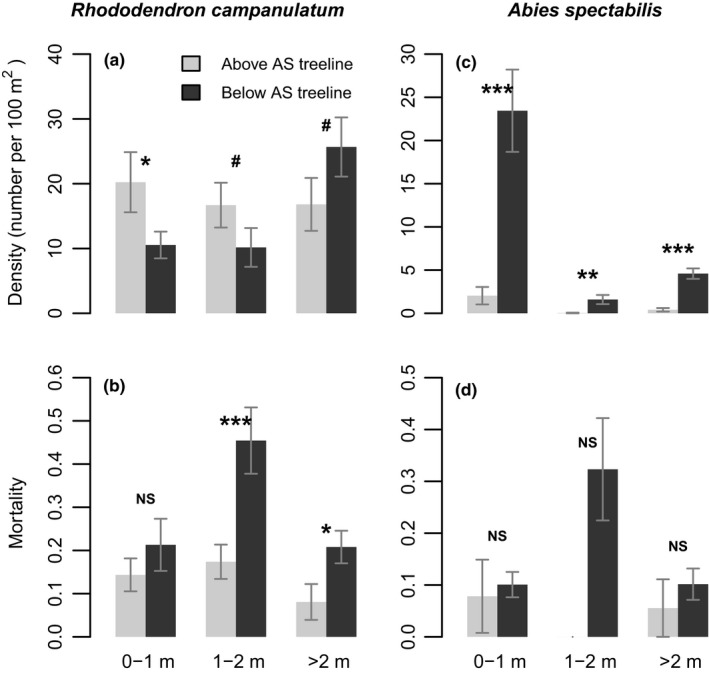
Density and mortality of *R. campanulatum* (left panels) and *A. spectabilis* (right panels) across treeline (mean ± 1 *SE*). Since there is spatial pattern of these measurements away from treeline (see Figures 2 and 3), an unbiased comparison of populations above versus below treeline requires sampling of similar spatial extent on the two sides of treeline. Among all the quadrats shown in Figures 2 and 3, some were discarded to maintain equal number of them on two sides of treeline of each transect. *p* value indicated as the following: *<.05; **<.01; ***<.001; #<.1; NS = not significant. *N* = 24–27 for density and mortality of *R. campanulatum* and density of *A. spectabilis*; *N* = 2–25 for mortality of *A. spectabilis*

Young *R. campanulatum* plants were significantly more numerous above treeline than below, while mature plants exhibited the opposite trend (Figure 4a). This difference between the distributions of the two size classes was itself significant: analysis of density by size class (0–1 m and >2 m classes only) by position (above or below the treeline) found a significant interaction (*p* = .02) indicating opposite trends in density across treeline for the youngest compared to mature plants of *R. campanulatum*. Mortality of >1 m tall *R. campanulatum* plants was higher below the treeline than above (Figure 4b). For younger plants (<1 m tall), position with respect to treeline was not significantly associated with mortality.

For *A. spectabilis*, the density of all age groups declined sharply right above the well‐defined treeline, with almost all the individuals confined to within 25 m of the treeline (Figure 2d–f). Contrary to abundance, mortality does not exhibit a clear pattern with elevation (Figure 3d–f). *Abies spectabilis* was significantly more abundant below treeline than above it in all size classes (Figure 4c). *Abies spectabilis* mortality across the treeline was not statistically significant (Figure 4d). However, the plants were so rare above treeline that significance would have been hard to achieve (Figure 2d–f). Despite the rarity of *A. spectabilis* above treeline, there was ample geographic space with good soil and nutrients (K. Mainali, unpublished).

### 3.3 Explanatory variables of density and mortality

**Figure 5:**
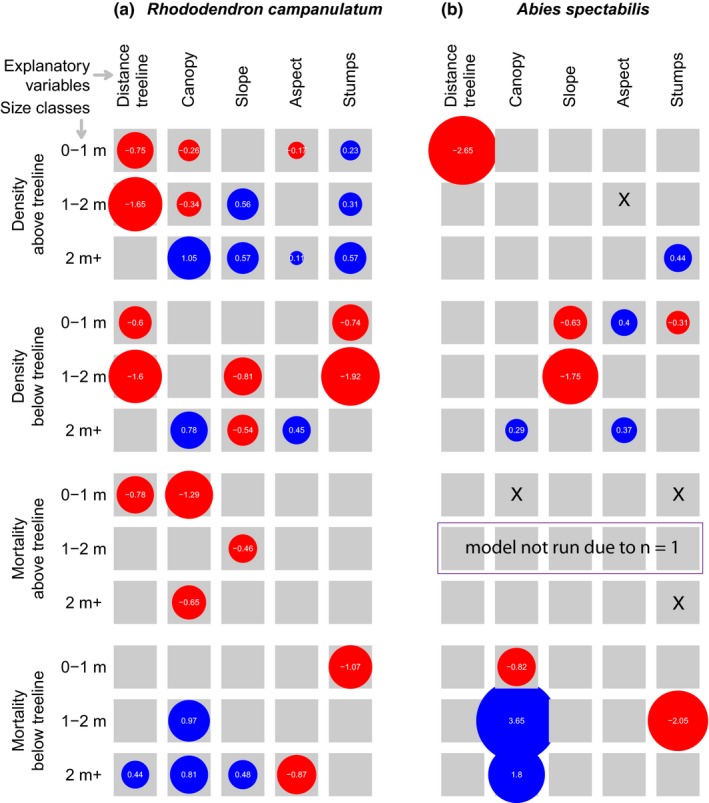
Potential explanatory variables of density and mortality, their effect size and direction of relationship for *R. campanulatum* (left panels) and *A. spectabilis* (right panels). The area of circle is proportional to effect size printed inside the circle. Positive relationships are shown in blue circles and negative in red ones. Only significant relationships at α of 0.05 are shown. Generalized linear mixed models (GLMM) were created with transect as random effect and up to five fixed effects shown as explanatory variables. The figure summarizes results of 24 separate GLMMs: 2 species × 2 dependent variables (density, mortality) × 2 sides of treeline (above, below) × 3 size classes. Each analysis consists of one response variable (either density or mortality) and five explanatory variables (arranged in the top row) which include the following: (1) Distance treeline as the elevation‐related variable: absolute elevational distance (vertical gradient) from treeline; (2) physical environment‐related variables: Slope (or, steepness), Aspect (see method for our approach of aspect calculation); (3) biological competition‐related variable: Canopy (visual estimation of the fraction of the quadrat that does not receive sunlight when sun is vertically above) as a measure of light availability and temperature and soil moisture‐related consequences; (4) anthropogenic disturbance: Stumps which measure the number of cut stumps in the plots. The analysis was performed using glmer() function of R package “lme4” with Poisson link function for density and binomial for mortality. GLMMs of mortality were weighted by density. Predictors marked with an “X” were removed from the model to achieve convergence. A GLMM for predicting mortality of 1–2 m size class of *A. spectabilis* above treeline could not be built as there was only one data point. Figure S1 shows another version of this figure with confidence interval of the effect size

Density of smaller plants of *R. campanulatum* was predicted by distance away from treeline on both sides of treeline; the density decreased similarly away from treeline in the studied belt of treeline ecotone, as depicted by comparable coefficients for above versus below treeline (Figure 5a). This effect was stronger for medium sized plants (coefficients = −1.6 to −1.65 when both variables were scaled in standardized units) than smaller ones (coefficients = −0.6 to −0.75) but the tall plants did not exhibit a statistically significant relationship with distance. Tree canopy provided by *A. spectabilis* strongly and positively related to density for tall *R. campanulatum* plants on both sides of treeline but canopy exerted a small negative effect on smaller plants above treeline. Steeper slopes supported denser patches above treeline but less dense patches below treeline. This trend was observed only for medium and tall plants. Aspect exhibited weak effect on density above treeline but below treeline, it had moderate positive effect on tall plants. For tall plants, aspect and canopy were significantly positively associated with abundance. The variable “Stumps” was positively related to abundance above treeline for all size classes (weak‐to‐moderate effect) but strongly negatively related to small and medium plants below treeline. The mortality of *R. campanulatum* has fewer significant predictors than abundance does. Small plants enjoy a lower mortality with distance upslope from treeline whereas tall plants have higher mortality with distance downslope from treeline. Canopy, in general, reduces mortality (denser shades linked to lower mortality) above treeline but increases the mortality below treeline. Steeper slopes decrease mortality of medium size class above treeline but increase mortality of tall plants below treeline. Aspect reduces mortality for tall plants below treeline (southern facing sunny flanks with lower mortality compared to those that receive less sunlight). Stumps are related to lower mortality of small plants below treeline.

Compared to *R. campanulatum*, *A. spectabilis* had fewer significant predictors of density and mortality (Figure 5b). Distance away from treeline had very strong negative influence on density of small plants. Canopy and aspect had weaker positive influences on densities of some of the size classes, but only below treeline. Steeper slopes supported thinner patches of the plants. “Stumps” had weak positive influence on density of taller plants above treeline and weak negative influence on density of small plants below treeline. For mortality, canopy and “stumps” were the only significant predictors. Canopy decreased mortality of small plants but increased that of medium and tall plants below treeline. “Stumps” were linked to lower mortality of medium plants below treeline.

### 3.4 Size–class distribution and regeneration potential

**Figure 6:**
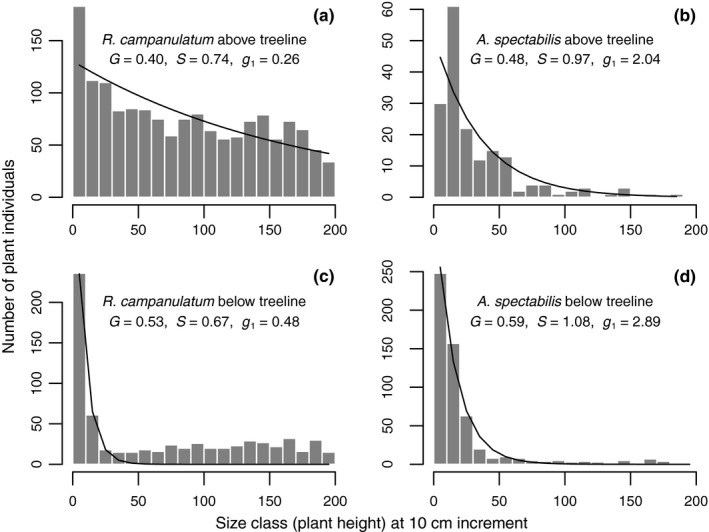
Size–class distribution of *R. campanulatum* (left panels) and *A. spectabilis* (right panels) above and below treeline. *G* = Gini index, *S* = Lorenz asymmetry index, *g*_1_ = coefficient of skewness. Because the exact height of plants >2 m tall was not measured, we excluded plants >2 m tall in this analysis. Line represents best fit of negative exponential to data: frequency = exp(*a* + *b* * plant size), *p* < .001 for both parameters *a* and *b* for all the plots. Estimates of the parameters not shown; *b* is always negative

Size–class distributions of both species on either side of the treeline (Figure 6) exhibited highly significant negative exponential regression (*p* < .01) between size classes and their frequencies in the studied plots. For both species, the skewness coefficient (*g*_1_) and Gini coefficient (*G*) were lower above treeline than below, indicating that there was reduced inequality in the Lorenz curve above treeline compared to below treeline. The *R. campanulatum* population below treeline had a more asymmetric Lorenz curve (*S* = 0.67) than the population above treeline (*S* = 0.74) indicating the presence of a relatively higher frequency of small size classes above treeline than below. The *A. spectabilis* population above treeline, with *S* < 1, also had inequality driven by higher frequency of small size classes than below treeline, with *S* > 1. All the three indices of skewness or inequality in the Lorenz curve (*G*, *S*, *g*_1_) indicate that both species had a higher fraction of younger individuals above treeline than below. However, a statistical test shows that the Lorenz curve was significantly different above versus below treeline only for *R. campanulatum* (two sample Kolmogorov–Smirnov test: *D* = 0.85, *p* < .001 for *R. campanulatum;* *D* = 0.24, *p* = .23 for *A. spectabilis*).

### 3.5 Relationship of height to age and circumference

**Figure 7:**
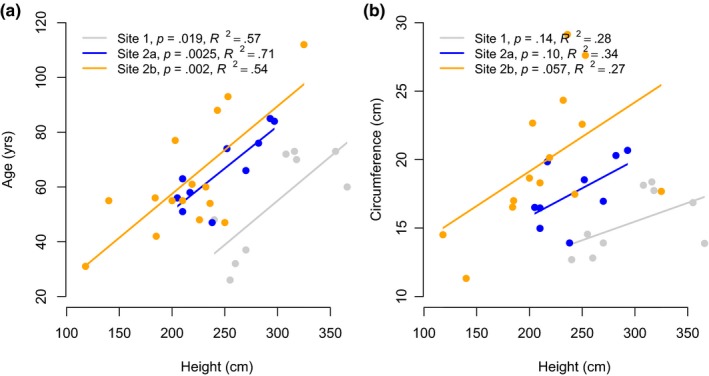
Relationship of plant height to age (left panel) and to circumference (right panel). Site 2 has two separate flanks which are shown separately

Height was linearly related to age and circumference in both study sites (Figure 7). The relationship was tighter between height and age (*R* ^2^ = .54–.71) than between height and circumference (*R* ^2^ = .27–.34. The coefficient of regression of age with height was identical across sites (Figure 7a) even though plant heights differed considerably between sites. Similar pattern (comparable coefficient, difference in extent of height) was observed in regression of circumference with height (Figure 7b).

## 4 DISCUSSION

As the climate continues to warm, temperature isotherms move uphill and low temperature constraints relax, generating more favorable conditions for tree establishment and growth at upper elevational range boundaries. This should alter the dynamics of forest regeneration by affecting reproduction and recruitment, ultimately manifesting as an upward shift in treeline. In fact, one of the best indicators of treeline sensitivity to climate warming is advance of seedlings above treeline (Holtmeier & Broll, 2005). Using analyses of size–class distributions, we found evidence for some level of regeneration of both species on both sides of the treeline, but with much greater regeneration of *R. campanulatum* than of *A. spectabilis* above treeline. In line with climate change expectations, we present evidence for a recent increased recruitment of *R. campanulatum* above the treeline and discuss evidence for the mechanistic drivers of the spatial patterns of density and mortality.

### 4.1 Stand demography and covariates

*Rhododendron campanulatum* in the 0–1 m and 1–2 m size classes had higher density above the treeline than below it; this trend was reversed for the >2 m size class, which showed a trend for higher abundance below the treeline but enjoyed lower mortality above it (Figure 4). Given the competition for light and the colder microenvironment at ground level below the treeline due to shading, it is not a surprise to see higher mortality of *R. campanulatum* saplings below treeline than above. Adult *R. campanulatum* plants that achieved a height of >2 m were, on average, ~50 years old (as determined with tree‐ring analysis), and therefore, germinated many decades ago. These individuals occurred up to 200 m above the treeline (Figure 2). In contrast, the response by *A. spectabilis* appears much less pronounced, with a weaker trend for age distribution to be increasingly skewed toward younger individuals at higher elevations. We found very limited evidence for expansion of *A. spectabilis* above treeline. All of the *A. spectabilis* size classes were almost exclusively confined to <25 m above treeline, with only a couple of sightings of small saplings beyond that point (Figure 2). The treeline comprised of old *A. spectabilis* trees essentially marked a hard boundary for this species (Figures 1 and 4c). As we observed, trees at this boundary were over a century old, indicating that this treeline position has been either static or contracting for over a century.

Density of both species responded mainly to absolute elevational distance from treeline; away from treeline, we observed lower density (significant result only for some size classes). Collectively, aspect, canopy, and distance away from treeline had similar effects on abundance of *R. campanulatum* on both sides of the treeline but slope and stumps had contrasting effects on the two sides of the treeline. Above treeline, stumps were positively associated with density of *R. campanulatum* but below treeline, the effect was negative and much stronger. The reason for this is not clear, but the positive association of stumps with density above treeline shows that selective tree‐felling has not been a substantial problem for *R. campanulatum* above treeline.

When a covariate has similar effect on the two sides of treeline, different size classes can still respond differently. For instance, denser canopy decreased density of small and medium *R. campanulatum* plants because these plants flourish in the sun. For taller plants, canopy increased density. Mortality of *A. spectabilis* decreased with canopy cover for <1 m plants and increased with canopy cover for >1 m plants, which is explained by the fact that young plants of *A. spectabilis* can grow in the shade (Shrestha, Ghimire, Lekhak, & Jha, 2007).

### 4.2 Regeneration potential across treeline in both species

It has long been recognized that the distribution of tree size classes in forests with sustainable regeneration takes a reverse J shape (Rao, Barik, Pandey, & Tripathi, 1990; West, Shugart, & Ranney, 1981). This type of distribution results from good reproduction and continuous recruitment (Bongers, Popma, Del Castillo, & Carabias, 1988). Therefore, a deviation from the reverse J shape indicates issues in recruitment. For instance, uni‐ or multimodal distribution indicates discontinuous recruitment and a flat line after the youngest size class indicates poor recruitment (Bongers et al., 1988). Both *R. campanulatum* and *A. spectabilis* populations above and below treeline strongly conformed to a negative exponential regression, indicating that both species are regenerating above and below treeline (Figure 6). However, *A. spectabilis* regeneration extended a much shorter distance above treeline, only around 25 m, compared to the 200 m elevational band above treeline in which *R. campanulatum* was regenerating.

Species at the treeline ecotone in other parts of the Himalaya have shown contrasting responses to climate change. Gaire, Koirala, Bhuju, and Borgaonkar (2014) observed upward shift of *A. spectabilis* in central Nepal Himalaya where another common tree species, *Betula utilis*, failed to shift upward in recent decades. Lv and Zhang (2012) studied *A. spectabilis* in the Mt Everest region of Tibet and found that it has had continuous recruitment below treeline over the last three decades, but only sporadic recruitment prior to that (i.e., >30 years ago). They also found that recruitment had a positive correlation with summer (June–Sept) temperature. Also, Gaire, Dhakal, Lekhak, Bhuju, and Shah (2011) reported that *A. spectabilis* has had substantial recruitment below treeline in recent decades near our study sites. These few studies showing *A. spectabilis* recruitment below treeline and upward shift of treeline are, however, contrasted by at least two studies in Nepal. Shrestha et al. (2014) reported a static treeline of *A. spectabilis* in the same general area of our site 1. Chhetri and Cairns (2015) recorded just a 22‐m upward shift of treeline in over a century.

### 4.3 Why the observations are likely a response to climate change

We present three lines of argument to support that our observations are driven by climate change, and not by other anthropogenic factors. First, we counted the number of cut stumps in each plot and found that logging was twice as frequent below treeline as above it: mean numbers of cut stumps per 100 m^2^ were 1.1 and 2.3, above and below treeline, respectively. Considering the long retention of stumps, these numbers represent historically low anthropogenic disturbance above treeline. In fact, the only users of the small trees were nomadic herders that stay in site 1 for about a month per year. Hence, cut stumps were entirely missing from site 2. A couple of kilometers from site 2 were two small huts with some cows and sheep that stayed there for half a year. Grazing pressure (visual estimation in the scale of 1–4, with 1 being not impacted at all and 4 being the highest among plots in arbitrary scale) was similar both above (1.9) and below (2.0) treeline.

Second, the responses of *R. campanulatum* that we observed fit nicely with globally observed treelines that are responsive to climate warming. Harsch and Bader (2011) found that globally, 80% of the diffuse treelines, which are characterized by gradual decrease in tree height, were found advancing upward because the primary driver of diffuse treeline is growth limitation enforced by growing season minimum temperature. This explains why *R. campanulatum*, growing luxuriantly with no sign of stunted growth at treeline, is regenerating fast above treeline in a wide space.

Third, there is spatial heterogeneity in anthropogenic disturbance affecting treeline ecotone dynamics at local scale. Such local drivers need to be evaluated on a case‐by‐case basis. Schickhoff et al. (2015) conclude that Langtang treeline ecotones are “near‐natural” (see their Figure 2) but affected by pastoral and forest user communities. Our field sites were situated very far (a day's walk) from human settlements, and several hours (site 1, 2 hr, and site 2, 5 hr) walk from tourist trails (not heavily used) and from isolated hotels. Schickhoff et al. (2015) also observed that north‐facing slopes are of lesser value to pastoral communities and support the vast majority of near‐natural treelines. Consequently, diffuse and less disturbed treelines are mostly confined to north‐facing slopes of the Himalaya (Chhetri & Cairns, 2015) as we observed in our both sites situated on north‐facing slopes. Given other researchers' general observations and our specific observation of minimal anthropogenic disturbance in the sites, we conclude that observed population dynamics of *R. campanulatum* across treeline is a response to climate warming. The failure of *A. spectabilis* to advance upwards indicates that upward advance of the treeline community will be species‐specific, as the requirements and tolerances of seedling establishment, growth, and survival are different for different species.

### 4.4 Constraints to tree recruitment at the upper elevation range boundary

Compared to below treeline, habitats above the treeline provide more light and, unintuitively, more shelter for tree seedlings. Increased shelter stems from the emergence of short vegetation (grasses and small bushes) above treeline. As a consequence, tree seedlings are both warmer and receive more sunlight when embedded in this short vegetation, thus numbers are often higher above than below the treeline (Körner, 2012). In contrast, animal browsing was limited and highly seasonal above treeline at our study sites, and other potential constraints of tree recruitment appeared to play minimal role.

The thick ground vegetation we observed above treeline likely favored greater recruitment of seedlings above than below treeline due to aerodynamic decoupling. That is, short shrubs, forbs, and grasses, when growing clumped, can decouple the local microenvironment from free atmospheric convection, resulting in substantial warming within and among vegetation clumps. Körner (2003) found temperatures within alpine shrub/grassland and within alpine cushion plants to be from 10°C to 20°C above ambient air temperature, respectively. Therefore, somewhat counter‐intuitively, saplings growing under mature tree canopy below treeline actually experience colder temperatures than those growing in the middle of short vegetation above treeline. As paradoxical as it sounds, Europe‐wide measurement of soil temperatures at 5 cm depth in 25 alpine sites showed that soil was, on average, 2°C warmer at 200–300 m above treeline than at the treeline itself (Körner, 2003). Thus, growing in clusters of short stature alpine vegetation offers beneficial facilitation to seedling recruitment above treeline by maintaining warmer air and soil temperatures (Körner & Paulsen, 2004). Facilitation from surrounding vegetation, that provides a warm microclimate for seed germination and growth, has been reported from several alpine areas (Anthelme, Cavieres, & Dangles, 2014; Choler, Michalet, & Callaway, 2001; Smith, Germino, Hancock, & Johnson, 2003).

As a sapling growing above treeline emerges above surrounding vegetation, the tip of the plant is exposed to ambient air temperature and the main axis fails to grow normally when ambient air is prohibitively cold. Compared to growth, photosynthesis is far less sensitive to low temperature, functioning at 50%–70% of full capacity at 5°C in cold‐adapted montane species (Körner, 2012). Consequently, in cold conditions, photosynthate is redirected toward accelerated growth of branches and girth toward the base resulting in higher ratio of basal diameter to plant height, called “taper.” This phenomenon has been confirmed in many species and in diverse mountain ranges of Alps, Kilimanjaro, Mexico, Bolivia (unpublished data of G. Hoch and F. Cohnen cited in Körner (2012). This dramatic increase in taper with elevation near treeline indicates that the upward growth of the main axis of a tree is more sensitive than trunk diameter growth to the particular adverse conditions encountered in that environment.

If the constraints of climate on taper that we discussed above were operating, then plant age and circumference should both show a concave curvilinear relationship with height, because as a plant continues to grow, progressively less plant height is added for a unit increase in age or circumference. Our data indicate no such relationship in *R. campanulatum* immediately above treeline (within ~50 m, Figure 7a,b). The relationships between height and age or between height and circumference appear to be linear, similar to those reported by Hörnberg, Ohlson, and Zackrisson (1995) for trees growing normally. Further, the two mountains in our study had surprisingly similar coefficients of regression in both of these relationships, suggesting that similar processes control growth pattern on the two mountains. These results indicate that all sizes of *R. campanulatum* have grown at a constant rate in recent decades, which suggests that ambient air temperatures have not been low enough to cause a tapered growth pattern typical of static high elevation treelines. Gamache and Payette (2004) observed a similar phenomenon, documenting a recent trend of increased shoot elongation at treeline in Canada and relating that to a warming during the 1990s.

If amelioration of temperature in recent decades is driving the observed patterns in density and mortality in *R. campanulatum*, then environmental conditions that strongly depend on elevation should be the important predictor(s) of the patterns. This proved to be the case: density of both species responds strongly to elevation‐related factors (Figure 5; effect size of “stumps” was shown to have little to no impact on both density and mortality), indicating that global drivers controlled density. In contrast, mortality of both species mostly responds to nonelevation‐related factors. The observed treeline dynamics are not likely to be responses to precipitation because precipitation in Langtang region has not shown any long‐term trend in the latter half of the 20th century (Shrestha, Wake, Dibb, & Mayewski, 2000; Shrestha et al., 2014). Therefore, density, but not mortality, is expected to change in a predictable fashion in a warmer future. These patterns of higher abundance, lower mortality and faster regeneration of *R. campanulatum* above treeline than below, and steady growth of plants above treeline indicate that an elevational range shift of *R. campanulatum*, most likely caused by warming, is currently underway.

### 4.5 Current status and future of treelines for *A. spectabilis* and *R. campanulatum*

Across the Nepal Himalaya, *A. spectabilis* and *Betula utilis* are the two most dominant tall tree species around treeline, *R. campanulatum* being the most dominant understory tree species (Liang, Dawadi, Pederson, & Eckstein, 2014; Stainton, 1972). In our extensive travel to various parts of high Himalayan systems (especially of BS), we observed that *A. spectabilis* is a late successional species with seedlings that can survive in deep shade. Stainton (1972), after his remarkable and extensive taxonomic expeditions across the Himalayas, concluded that “typically” *A. spectabilis* is not an early successional species. At elevations where *R. campanulatum* and *A. spectabilis* both grow, *R. campanulatum* is an early successional plant, requiring sunlight for seedling establishment. It is a common observation that *R. campanulatum* makes a pure stand above the limit of *A. spectabilis*. Below the pure stand and treeline, *R. campanulatum* grows under the canopy of tall trees of either *A. spectabilis* or *B. utilis* or mixed stands of both. We expect that as *R. campanulatum* continues to grow above treeline at our study sites, it will be able to cast shade that will effectively eliminate ground vegetation as seen under taller stands of *R. campanulatum* in the field sites, providing suitable habitat for *A. spectabilis* to establish; we observed many cases of *A. spectabilis* saplings growing under deep shade of *R. campanulatum* and other species.

We predict this trajectory of succession based on (a) our finding that canopy cover decreases *A. spectabilis* mortality for short plants but increases mortality for tall plants and (b) other studies that show abrupt treelines (like the boundary of *A. spectabilis*) form primarily because of drastically low seedling survival and growth as a result of frost, wind, and permafrost (Harsch & Bader, 2011), highlighting the importance of facilitation from surrounding plants that provide shelter from harsh climatic conditions (Armand, 1992; Batllori, Camarero, Ninot, & Gutiérrez, 2009).

Current population dynamics of young plants above treeline can indicate the future potential of treeline shift (Hofgaard et al., 2009). We predict that a pure stand of tall *R. campanulatum* will form over the next a few decades above the treeline. Over time, we expect young individuals of *A. spectabilis* to project above the low canopy of *R. campanulatum* and ultimately increase shading that would cause decline in the population of *R. campanulatum*. Low‐statured *R. campanulatum* is likely to be replaced by tall *A. spectabilis* trees over time, eventually shifting forest community structure.

## 5 CONCLUSIONS

Globally, both abrupt treelines, characterized by a sharp boundary of tall trees giving way to low alpine vegetation, and krummholz treelines, characterized by stunted and deformed trees at the margin, have mostly failed to respond to climate warming with only a quarter of them advancing (Harsch et al., 2009). For the Himalaya, which is projected to experience higher than global average increase in temperature (IPCC, 2007), we document evidence for a recent surge in forest growth above treeline by one of our study species, *Rhododendron campanulatum;* forest stands above treeline were regenerating faster than below treeline, having both lower mortality and higher recruitment. *Rhododendron campanulatum* plants above treeline appear to have had continuous growth (as opposed to exhibiting a Krummholz zone of stunted plants). Mortality of young *R. campanulatum* plants above treeline, often nested in a low stature deciduous Rhododendron carpets, was not determined by elevation‐related variables, but that of mature plants was. This provides support to the hypothesis that facilitation was important for young plants and temperature amelioration for mature plants. *Rhododendron campanulatum* is already shifting upward away from the *Abies* treeline. Conversely, *A. spectabilis* forms a very conspicuous treeline of tall trees that are over a century old right at the edge of treeline. This treeline has been stable for over a century. It has failed to advance upward which aligns with the following observations: (a) its nature as late successional species, (b) elevation almost never being a significant predictor of abundance, (c) its confinement to ~25 above treeline, (d) close to zero survival of plants beyond seedling and small saplings above treeline, and (e) importantly, canopy being an important predictor of mortality: canopy decreases mortality for <1 m tall plans but increases mortality for >1 m tall plants, indicating that small plants of *A. spectabilis* are shade tolerant and should be able to advance in elevation under cover of the expanding *R. campanulatum*. Hence, we predict that, although the *A. spectabilis* treeline has been stable till now, it will gradually overtake the current luxuriantly growing patches of *R. campanulatum* above treeline and a new treeline, dominated by *A. spectabilis*, will form over the coming century above the current *A. spectabilis* treeline.

## CONFLICT OF INTEREST

None declared.

## AUTHOR CONTRIBUTIONS

K. M. conceived the ideas; K. M., C. P, and B. S. designed methodology; K. M., B. S., and R. S. collected the data; K. M., E. G., A. A., M. S., and C. P. analyzed the data; K. M. wrote the first draft of the manuscript, and M. S. and C. P. revised it. All authors contributed critically to the drafts and gave final approval for publication.

## Data Availability

All data and script for analysis and plotting are available at Dryad with the following link: https://doi.org/10.5061/dryad.nzs7h44n2
