# Supplementary figures and images for: Contrasting responses to climate change at Himalayan treelines revealed by population demographics of two dominant species
Source: Ecol Evol. 2020 Jan 28;10(3):1209–22. doi: 10.1002/ece3.5968 (PMC7029064; doi:10.1002/ece3.5968)

**(a) Rhododendron density**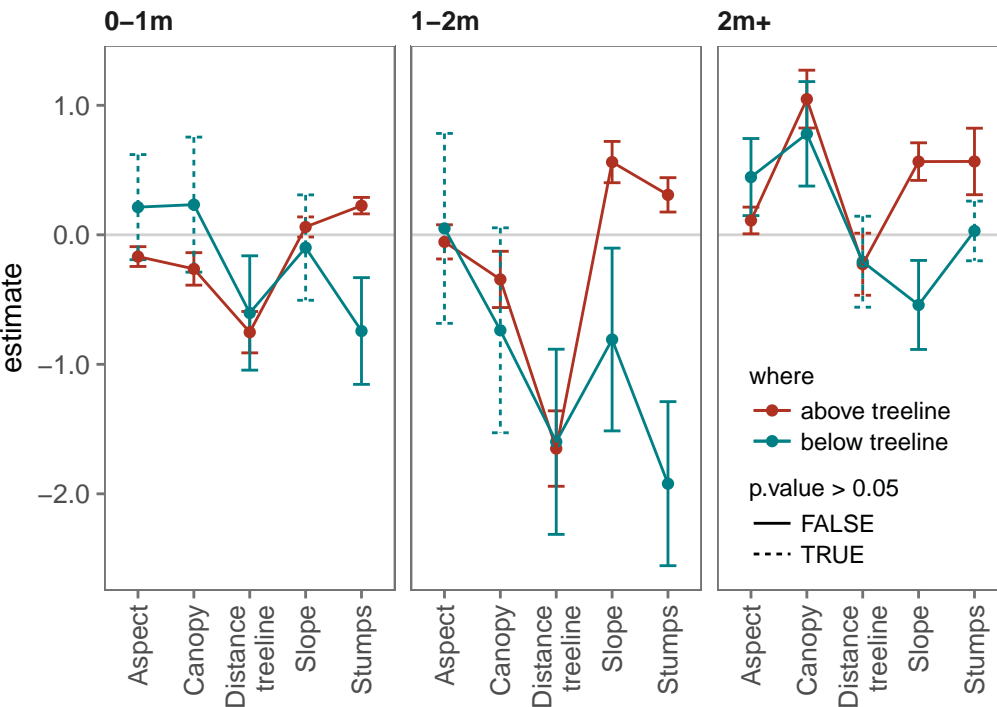**(b) Abies density**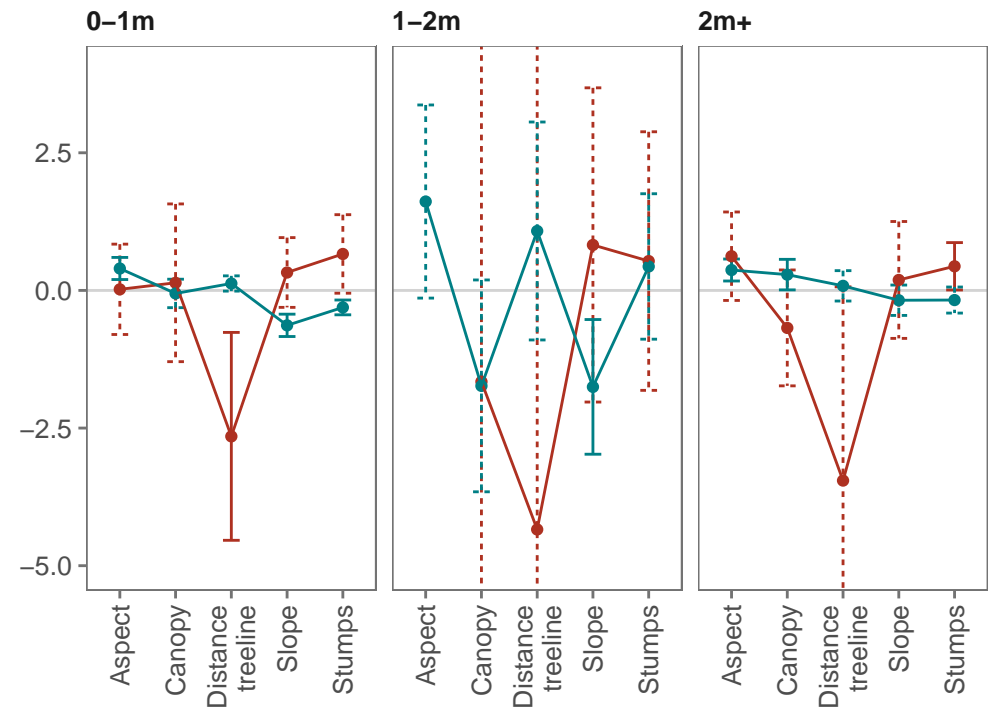**(c) Rhododendron mortality**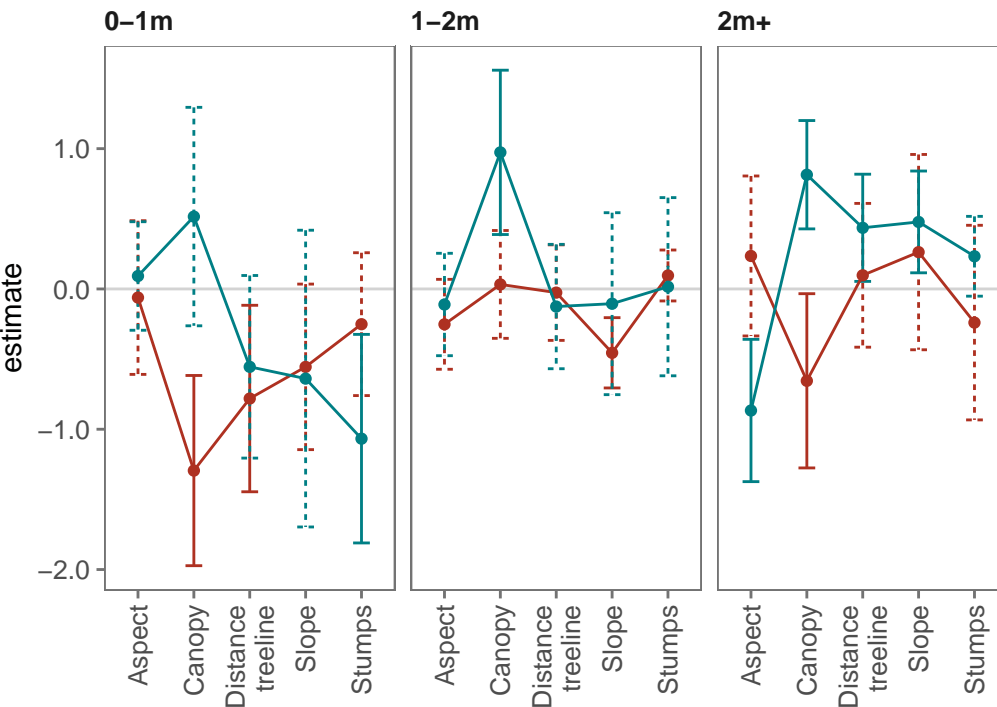**(d) Abies mortality**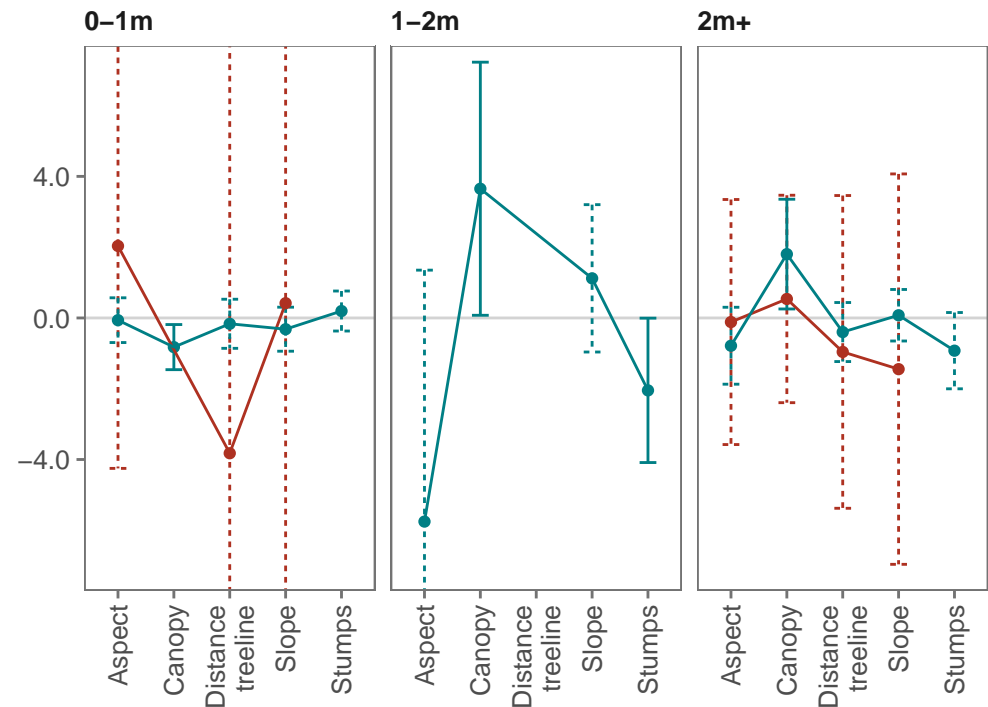

Supplement: Supplementary file 1 [file ECE3-10-1209-s001.pdf]
